# Peripheral blood and bronchoalveolar leukocyte profile in lung transplant recipients and their changes according to immunosuppressive regimen: A single‐center experience

**DOI:** 10.1002/iid3.673

**Published:** 2022-07-12

**Authors:** Zsuzsanna Jáky‐Kováts, Melinda Vámos, Zsolt István Komlósi, András Bikov, Ildikó Madurka, Gergő Szűcs, Veronika Müller, Anikó Bohács

**Affiliations:** ^1^ Department of Pulmonology, Faculty of Medicine Semmelweis University Budapest Hungary; ^2^ Department of Genetics, Cell‐ and Immunobiology, Faculty of Medicine Semmelweis University Budapest Hungary; ^3^ Division of Infection, Immunity & Respiratory Medicine University of Manchester Manchester UK; ^4^ Department of Thoracic Surgery, Faculty of Medicine Semmelweis University Budapest Hungary; ^5^ Department of Thoracic Surgery National Institute of Oncology Budapest Hungary

**Keywords:** acute cellular rejection, bronchoalveolar lavage, differential diagnosis, lung transplant, PBMC

## Abstract

**Background:**

After lung transplantation (LuTX), lower respiratory tract infections (LRTI) and acute cellular rejection (ACR) are associated with changes in peripheral blood and bronchoalveolar lavage fluid mononuclear cell profile (PBMC and BALIC). PBMC is also influenced by immunosuppressive regimen and its changes with postoperative time. First‐year PBMC and BALIC changes were evaluated in this study with rabbit anti‐thymocyte globulin (ATG) and alemtuzumab (AL) induction therapy.

**Methods:**

In total, 64 LuTX recipients were included, 53 of them received AL and 11 ATG as induction therapy. PBMC and BALIC were examined routinely and in cases suspicious of infection and/or rejection. A PBMC‐ and BALIC‐based algorithm for infection and rejection prediction was also tested.

**Results:**

In the AL group, peripheral blood lymphocyte and basophil cell numbers were significantly reduced, while the neutrophil cell number elevation during LRTI was significantly higher compared to the control. Early postoperative measurements showed a lower BALIC lymphocyte count. The algorithm had 17% sensitivity and 94% specificity for ACR in all patients and 33% sensitivity and 95% specificity for ACR with coexisting LRTI.

**Conclusion:**

BALIC is not significantly influenced by the immunosuppressive regimen. PBMC‐ and BALIC‐based algorithm may improve the differential diagnosis of ACR.

## Graphical abstract

Bronchoalveoar lavage fluid cytology is not significantly influenced by the immunosuppressive regimen after lung transplantation. Peripheral blood mononuclear cell and bronchoalveolar lavage fluid cytology analysis based on the algorithm described by Speck et al. may improve the differential diagnosis of ACR.

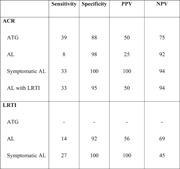

## 1 INTRODUCTION

In recent decades, the number of lung transplants (LuTX) has been rising,^1^ and survival rates tend to improve. Despite major developments in therapeutic possibilities, first‐year mortality remains high.^1^ After the early postoperative period, the two major complications are infections, especially lower respiratory tract infections (LRTI), and acute cellular rejection (ACR).^1^, ^2^ The gold standard diagnoses are microbiological samples of the lower respiratory tract and transbronchial biopsy (TBB) histology. Numerous studies aimed to find biomarkers or determine scoring systems using less invasive methods. Bronchoalveolar lavage fluid (BALF) examinations, including differential cytology, are promising with elevated neutrophil,^3^ eosinophil,^4^ or lymphocyte count during rejection. A recent study also described a literature‐based differential diagnostic algorithm using BALF and peripheral blood cytology.^5^ In an earlier study, added to BAL cytology, total BAL cell Trx levels were shown to be elevated in rejecting patients compared to the nonrejecting group.^6^ However, induction therapy and immunosuppression protocols have high variability between the transplant centers and over time, which makes comparing and synthesizing the results difficult. To our knowledge, the possible effects of differences in the immunosuppressive regimen on bronchoalveolar lavage immune cells (BALIC) had not been examined earlier.

In the last decades, one of the successfully introduced immunosuppressive methods is induction therapy. Antithymocyte globulin (ATG) and IL‐2 antagonist basiliximab followed by a monoclonal anti‐CD52 antibody, alemtuzumab, are the most commonly used drugs for this purpose. A recent study described patients with ATG induction as having lymphocyte counts close to the values of control LuTX patients who received no induction therapy,^7^ and ATG was shown earlier to have less effect on the lymphocyte number in BALIC than basiliximab.^8^ Administering alemtuzumab also results in significant lymphocyte depletion. B‐lymphocyte subsets recover after 3−12 months, while CD4+ and CD8+ T cells may be depleted in as long as 36 months.^9^ CD52 is also expressed in comparatively smaller amounts on macrophages, monocytes, and eosinophils^10^, ^11^; thus, changes in the count of these cell types are also possible. Alemtuzumab has been proven to be safe and useful in the prevention of acute rejection,^12^ also improving bronchiolitis‐obliterans syndrome‐free survival,^13^ while infective complication possibilities are similar to those following the standard immunosuppressive regimen.^14^

As—to our knowledge—no data have been published on peripheral blood mononuclear cells (PBMC) and BALIC profiles after alemtuzumab induction therapy. In this study, the time course and the possible differences compared to ATG induction were examined.

In the second part of the study, PBMC and BALIC data were analyzed with the literature‐based differential diagnostic algorithm described by Speck et al.^5^ The algorithm uses PBMC and BALIC changes to assess the possibility of ACR and was generated from earlier literature data. The present study aimed to determine whether the algorithm could be a minimally invasive differential diagnostic tool for the ACR of the lung allograft.

Therefore, our aim was to determine the BALIC changes in LuTX recipients after alemtuzumab induction therapy. LuTX patients who received ATG as induction therapy served as a control group. We also tested the real‐life reliability of the recently described algorithm,^5^ which uses PBMC and BALIC analysis for the diagnosis of LRTI and ACR after LuTX.

## 2 METHODS

### 2.1 Study design

This retrospective case‐series study analyses data recorded in the standard medical documentation of lung transplant recipients who received routine posttransplant care at our department.

### 2.2 Study population and immunosuppressive regimen

In total, 64 Hungarian LuTX patients in postoperative care were included in the study, who all underwent double LuTX. Postoperative care after discharge from the Department of Thoracic Surgery was provided for all patients at the Department of Pulmonology at Semmelweis University, Faculty of Medicine. LuTX was performed between February 2011 and November 2016, except for two patients who were transplanted in 2006. The patients received either alemtuzumab (AL group) or ATG as induction therapy at the surgical intensive care unit. In the AL cases, LuTX was performed between February 2012 and November 2016, and in the ATG, cases between 2011 and 2012, except for two patients who received lung transplants in 2006. The main immunosuppressive regimen in the ATG group was a combination of tacrolimus, mycophenolate, and prednisolone, though one ATG patient received cyclosporine A instead of tacrolimus. Patients in the AL group received lower doses of tacrolimus and prednisolone, while mycophenolate therapy was started between the 6th and 15th postoperative months of recovery from peripheral blood lymphopenia. Mycophenolate treatment was paused or was administered in lower doses in the case of neutropenia (absolute neutrophil count <2 G/L). All of the patients received CMV and Aspergillus prophylaxis for the first 3 postoperative months and lifelong Pneumocystis carinii prophylaxis.

### 2.3 Study variables

Regular patient check‐ups included the registration of complaints and symptoms, physical examination, chest X‐ray or computer tomography, and clinical laboratory measurements including differential blood cell analysis, pulse oximetry, and lung function measurements. These data assisted the diagnosis of ACR and LRTI. Surveillance bronchoscopy with TBB for histology and BAL for microbiological analysis and mononuclear cell profiling has been regularly performed after 4, 8, and 12 weeks, and at 6 and 12 months following engraftment in all patients. Bronchoscopy including BAL and TBB was also performed if symptoms of possible rejection were observed, such as a decline of forced expiratory flow between 25% and 75% of forced vital capacity (FEF25−75), which was not accompanied by symptoms of LRTI or did not respond to adequate antibiotic therapy. TBB was not done if a large volume of purulent mucus was present. Both surveillance and clinically indicated urgent bronchoscopy results were included into the analysis, and blood samples for PBMC analysis were collected on the day before bronchoscopy.

### 2.4 Bronchoscopic procedure and analysis of the BALF

For BAL, usually 120 ml of 0.9% saline was instilled in 40 ml fractions, then suctioned at 0.2 mBar.

A part of the recovered fluid was sent to microbiological analysis, and 15 ml BALF was used for cytological analysis. After preparation, the slides were analyzed by photomicroscope and the cell percentages from this examination were included in the analysis. The BALIC absolute cell counts were calculated from the same cell percentages; thus, BALIC leukocyte counts were defined as the product of the proportion of leukocytes in the BALF, and the mononuclear cell count was determined by the blood cell counter device.

### 2.5 Definition of infection and rejection

LRTI were defined according to the International Society of Heart and Lung Transplantation consensus criteria.^15^ However, for patients who were treated with antibiotics, a recent positive culture—with symptoms and laboratory results concordant with an acute infection—was defined as an infection only if it was accompanied by current culture positivity. ACR was defined according to the standard nomenclature based on TBB specimens.^16^ The patients were considered to be stable if they had no infection, ACR, or any other verified graft dysfunction.

### 2.6 Clinical test of the diagnostic algorithm

The sensitivity, specificity, and positive and negative predictive values (PPV and NPV) of the algorithm described by Speck et al. were determined for the control and AL patient groups. As the tests were performed before the loss of FEV1 reached 10%, only the PBMC and BALIC criteria were used. As the cell percentages calculated by the total mononuclear cell counts were used in the development of the algorithm, these cell percentages were used for testing the sensitivity and specificity of the algorithm in our study as well. Table 1 summarizes the PBMC and BALIC criteria used in our analysis for LRTI and ACR, as published previously by Speck et al.^5^

**Table 1:** Cellular changes used as indicators for LRTI or ACR

	LRTI	ACR
PBMC		
Neutrophils	>9 G/L	
Lymphocytes		>1.5 G/L
Eosinophils		>0.04 G/L
Basophils		>2%
BALIC		
Neutrophils		>12%
Lymphocytes		>20%
Eosinophils		Present
Basophils		>2%

Abbreviations: ACR, acute cellular rejection; BALIC, bronchoalveolar lavage fluid immune cells; LRTI, lower respiratory tract infection; PBMC, peripheral blood mononuclear cells.

### 2.7 Statistical analysis

Comparisons were made according to induction therapy, and between the stable, infection, and rejection subgroups during and after the first 6 postoperative months. The changes according to postoperative time were analyzed by comparing the time periods. *P*_1−3_ lasted from the 14th day until the end of the 3rd month, *P*_4−6_ from the 4th to the end of the 6th month, and *P*_7−13_ from the 7th to the 13th. Statistical analysis was performed with IBM Statistical Package for Social Sciences version 25 software (SPSS Inc.). The comparisons between the two subgroups of induction were made using unpaired *t* test, Fisher's exact, and Mann−Whitney *U*. The postoperative changes within the three time periods were analyzed by Kruskal−Wallis, and Bonferroni tests. Data are represented as mean ± SD and interquartile ranges. *p* < .05 was considered a significant difference between groups.

The data were retrospectively analyzed from patient charts. The study was approved by the ethical committee of Semmelweis University (258/2013).

## 3 RESULTS

### 3.1 Patient characteristics

Out of the 64 patients, 53 received 30 mg alemtuzumab as induction therapy (AL group), while the other 11 patients with ATG induction therapy served as the control group. Detailed patient characteristics are shown in Table 2

**Table 2:** Patient characteristics, underlying diseases, and induction therapy

	Lung transplanted patients (*n* = 64)			
	ATG (*n* = 11)	Alemtuzumab (*n* = 53)	*p*	
Age at transplantation in years (mean ± SD (range))	47.22 ± 9.17 (32−63)	44.31 ± 14.49 (17−64)	ns	
Gender			ns	
Male	7 (64%)	27 (51%)		
Female	4 (36%)	26 (49%)		
Underlying disease			ns	
CF	2 (18%)	16^a^ (30%)		
COPD	4 (36%)	15 (29%)		
IPF	2 (18%)	10 (19%)		
PAH	1 (9%)	9^a^ (18%)		
LAM	2 (18%)	1 (2%)		
Sarcoidosis	—	1 (2%)		

Abbreviations: CF, cystic fibrosis; COPD, chronic obstructive pulmonary disease; IPF, idiopathic pulmonary fibrosis; LAM, lymphangioleiomyomatosis; PAH, pulmonary arterial hypertension; reTX, retransplantation.

^a^ Including 2 reTX

### 3.2 Prevalence of infection and rejection

The study included 135 BALF and blood samples from the 53 AL patients, and 24 from the 11 ATG patients. Infection was present in 37% of the AL cases and in 33% of the control cases (OR: 1.250, 95%CI: 0.48−3.28, ns), while ACR was found in 9% and 33%, respectively (OR: 0.24, 95% CI: 0.08−0.75, *p* = .013).

Nearly all ACRs were A1 rejections, with one A2 rejection in the AL and two A2 rejections in the ATG group. Seven infections in the AL group required hospitalization with three patients needing intensive care, based on both infection and chronic lung allograft dysfunction. In the ATG group, two patients were hospitalized during LRTI, one of them because of concomitant A2 rejection and need for parenteral therapy, while the other patient had a nonsevere LRTI in the early postoperative period before the first emission after the transplantation.

### 3.3 Clinical usefulness of the diagnostic algorithm

In the AL group, in 35 cases from the 135 (26%), the patients had symptoms of LRTI or lung function decline. Among the symptomatic patients, 3 had ACR, and 22 of them had LRTI. 75% of all ACR and 55% of all LRTI in this group were asymptomatic; therefore, the algorithm was also tested as a screening examination. Sensitivity, specificity, and positive and negative predictive values of the algorithm for different subgroups are summarized in Table 3.

**Table 3:** Reliability of the algorithm

	Sensitivity	Specificity	PPV	NPV
ACR				
ATG	39	88	50	75
AL	8	98	25	92
Symptomatic AL	33	100	100	94
AL with LRTI	33	95	50	94
LRTI				
ATG	—	—	—	—
AL	14	92	56	69
Symptomatic AL	27	100	100	45

Abbreviations: ACR, acute cellular rejection; AL, patient group with alemtuzumab induction therapy; ATG, patient group with thymoglobulin induction therapy; LRTI, lower respiratory tract infection; NPV, negative predictive value; PPV, positive predictive value.

### 3.4 Differences in peripheral blood and BALF immune cell parameters

**Figure 1:**
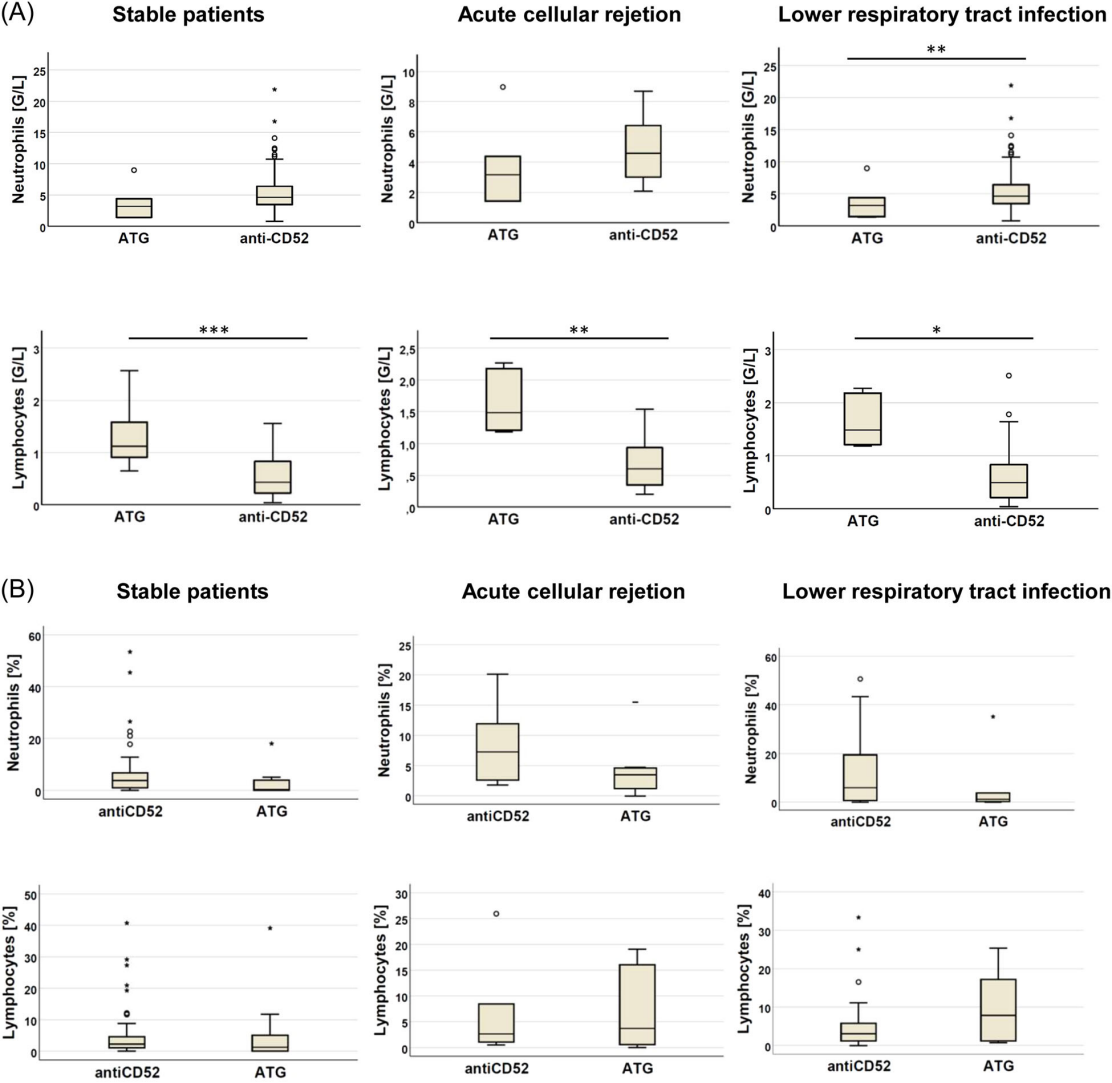
(A) Differences in peripheral blood neutrophil and lymphocyte cell counts according to induction therapy in stable patients, in acute rejection, and in lower respiratory tract infections. Lymphocyte counts were shown to be lower after anti‐CD52 induction therapy, while the elevation in the neutrophil cell count was more pronounced in this group compared to the results after induction with ATG. (B) Comparison of bronchoalveolar lavage fluid neutrophil and lymphocyte cell counts according to induction therapy in stable patients, in acute rejection, and in lower respiratory tract infections. No significant differences were found. ATG, anti‐thymocyte globulin. *p < .5, **p < .01, ***p < .001.

According to the induction therapy, in stable patients, no significant differences were observed in BALIC. PBMC lymphocyte cell counts were lower in the AL group compared to the ATG group (0.027 ± 0.047 vs. 0.066 ± 0.049 G/L, *p* = .001), and neutrophil cell count was higher (5.14 ± 3.12  vs. 3.65 ± 1.58 G/L, *p* < .01) if investigating the patients in stable condition. In samples of patients with ACR, PBMC lymphocyte count was lower (0.67 ± 0.49 vs. 1.64± 0.48 G/L, *p* < .01) than in the ATG group. PBMC differences in LRTI were similar to the differences observed in stable patients, with lower lymphocyte (0.596 ± 0.531 vs. 1.03 ± 0.38, *p* < .05) and higher neutrophil count (5.69 ± 3.45 vs. 2.56 ± 2.30, *p* < .001). White blood cell count was also higher in LRTI in the AL group (6.81 ± 3.74 vs. 3.95 ± 2.62, *p* < .005) (Figure 1). Detailed results are summarized in Supporting Information: Table S1.

### 3.5 Changes in peripheral blood and bronchoalveolar lavage immune cell profiles in light of postoperative time

**Figure 2:**
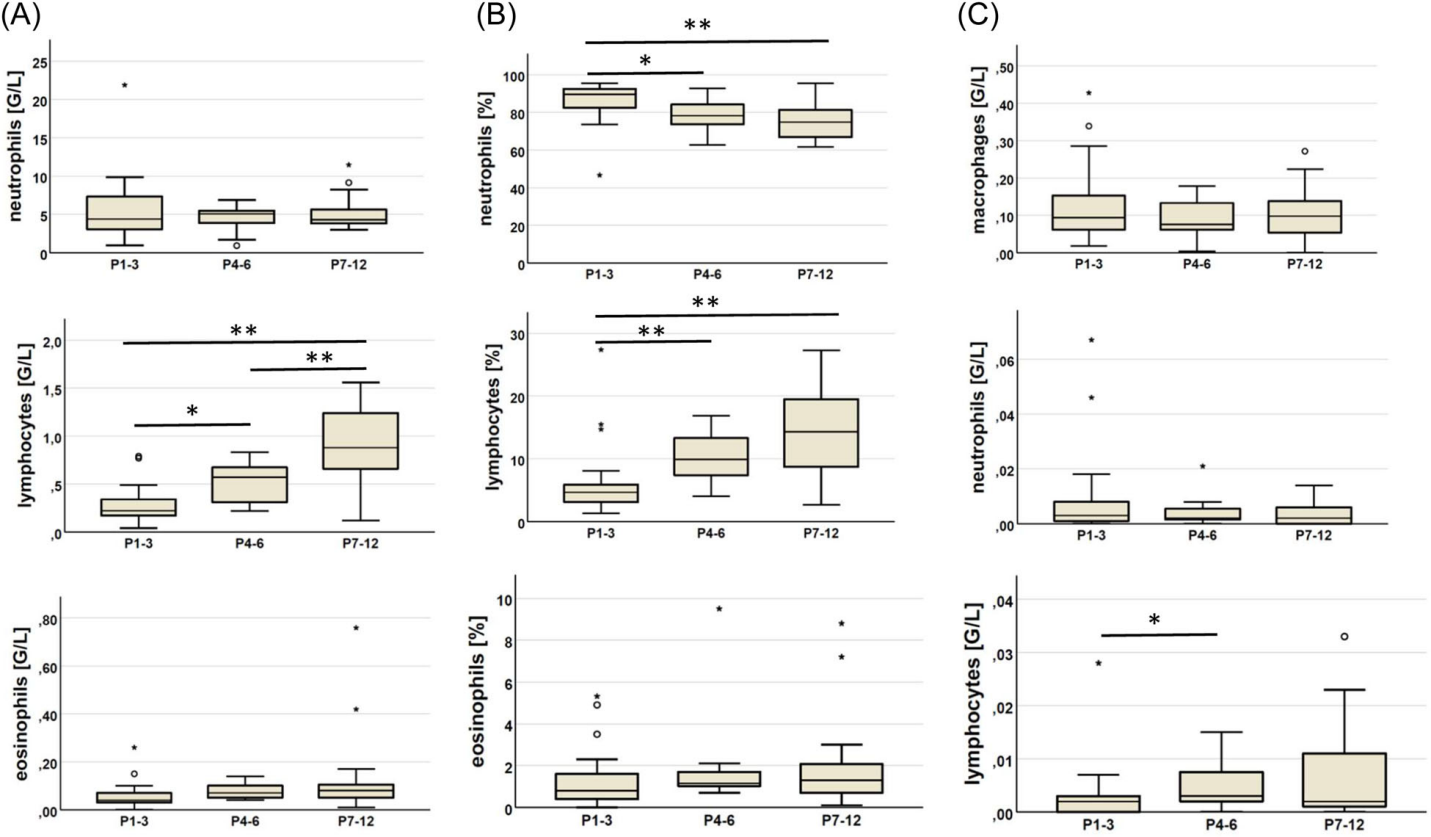
Changes in peripheral blood neutrophil, lymphocyte, and eosinophil cell count (A) and cell percentages (B) and bronchoalveolar lavage fluid macrophage, neutrophil cell, and lymphocyte count (C) in stable patients according to postoperative time periods. *P*_1−3_, *P*_4−6_, *P*_7−12_: the periods from the 1st to the 3rd, from the 4th to the 6th, and from the 7th to the 12th postoperative month, respectively. *p < .05, **p < .01.

The data from the stable patients of the AL group were analyzed according to the postoperative time periods. In PBMC, a significant rise was observed in lymphocyte cell counts, while the neutrophil percentage was declining. In BALF, absolute lymphocyte count was significantly lower in the first three postoperative months (Figure 2). Detailed data are shown in Supporting Information: Table S2.

## 4 DISCUSSION

The asymptomatic ACRs found in this study were mostly A1 rejections, and are therefore clinically less relevant, as the need for treatment is not obvious in low‐grade rejection in the absence of clinical signs and symptoms.^17^ On the other hand, minimal rejection can be persistent, and it can lead to the development of higher grade rejection^18^ or bronchiolitis obliterans syndrome,^19^ especially in cases detected via nonsurveillance examinations.^20^ A more important finding was that more than 50% of definitive LRTIs in LuTX recipients were only detected by surveillance bronchoscopies, based on positive BALF culture and endobronchial signs of inflammation.

In stable AL LuTX recipients, investigating changes in PBMC according to postoperative time yielded results as expected with increasing lymphocyte cell counts over time. Although the complement‐dependent cytolytic effect of alemtuzumab on neutrophils was also described earlier,^21^ in this study no significant changes in the absolute neutrophil count were detected. The significant lowering of neutrophil percentage may result from the recovery of other leukocyte counts and from the introduction of mycophenolate mofetil in the second half‐year in some of the patients. The only significant change in BALIC was the significantly higher absolute lymphocyte count in *P*_4−6_ compared to *P*_1−3_, which may be explained by the decreasing effect of alemtuzumab. After the sixth postoperative month, the BALIC results of the AL group showed no significant changes in stable patients. There were also no significant differences between the AL and ATG groups during ACR or LRTI. These findings may suggest that BALIC data could be comparable between patients with different immunosuppressive regimens.

Isolated subgroups of patients with infection or rejection according to the postoperative time periods were not investigated because of the low number of these events in the study population.

Regarding the usefulness of the algorithm, the action mechanism of alemtuzumab may be responsible for the very low sensitivity observed after induction therapy regarding peripheral lymphocytosis, due to lymphopenia caused by the anti‐CD52 antibody. It should be noted that sensitivity and specificity are also influenced by the fact that ACR was less prevalent in the AL patient group than in the ATG group. Furthermore, the specificity of the algorithm for ACR was not influenced by the presence of an LRTI, and in these cases, the sensitivity was found to be higher. Therefore, the algorithm could help the early diagnosis of ACR in cases with coexisting LRTI. On the other hand, a high negative predictive value can help rule out ACR.

Of course, our study had several limitations. It should be noted that bacterial, fungal, and viral infections were not differentiated, although isolated fungal or viral infections were rare. Furthermore, cases of chronic allograft dysfunction were also included in the study which may affect cytologic results.

Great diversity was observed in PBMC and BALIC in stable LuTX patients and in the ACR or LRTI subgroups, which may be partly due to overlapping cases of infection with rejection.

It should be noted that after alemtuzumab treatment, lower doses of tacrolimus and prednisolon were administered, and mycophenolate was introduced only after 6 months; thus, differences in the immunosuppressive regimen may interfere with the effects of the induction therapy.

## 5 CONCLUSIONS

The BALF immune cell‐based algorithm tested here may serve as a useful complementary method in clinical decision‐making in symptomatic patients, parallel to the standard determination of ACR, especially in ACR concomitant to an LRTI. Surveillance bronchoscopies were shown to have an important role, as more than half of the infections and rejections were asymptomatic and were detected by regular check‐up examinations including bronchoscopy. The diversity of leukocyte changes in peripheral blood and BALF found in this study in patients with ACR could signal different pathomechanisms beyond ACR. As there were no significant differences in BALIC in terms of immunosuppressive regimen, data of centers with different protocols could be compared.

## AUTHOR CONTRIBUTIONS

Zsuzsanna Jáky‐Kováts, Veronika Müller, Ildikó Madurka, and Anikó Bohács performed the patient examinations, Gergő Szűcs and Zsolt István Komlósi made the cytologic analysis, Zsuzsanna Jáky‐Kováts, András Bikov, Veronika Müller, and Anikó Bohács designed the research study; Zsuzsanna Jáky‐Kováts and Melinda Vámos aquisited the data; Zsuzsanna Jáky‐Kováts, Melinda Vámos, and András Bikov analyzed the data; Zsuzsanna Jáky‐Kováts and Anikó Bohács wrote the paper; and all authors approved the final manuscript.

## CONFLICT OF INTEREST

The authors declare no conflict of interest.

## Supporting information

Supporting Information.

Supporting Information.

## Data Availability

The data that support the findings of this study are available on request from the corresponding author. The data are not publicly available due to privacy or ethical restrictions.
