# Supplementary material for: Peripheral blood and bronchoalveolar leukocyte profile in lung transplant recipients and their changes according to immunosuppressive regimen: A single‐center experience
Source: Immun Inflamm Dis. 2022 Jul 12;10(8):e673. doi: 10.1002/iid3.673 (PMC9274796; doi:10.1002/iid3.673)
Supplement: Supplementary file 1 — Supporting Information. [file IID3-10-e673-s001.docx]

|  | **PBMC (G/L)**  **mean**±SD | | | **BALIC (%)**  **mean**±SD | | |
| --- | --- | --- | --- | --- | --- | --- |
| **All samples** | **Control**  **(n=22)** | **Alemtuzumab**  **(n=135)** | **p** | **Control**  **(n=22)** | **Alemtuzumab (n=135)** | **p** |
| Lymphocytes | 1.30±0.54 | 0.584±0.455 | <0.001 | 7.74±10.24 | 6.29±9.91 | ns |
| Eosinophils | 0.064±0.046 | 0.087±0.123 | ns | 0.704±1.873 | 0.510±1.755 | ns |
| Neutrophils | 3.43±2.14 | 5.12±3.19 | <0.01 | 4.49±8.36 | 9.71±14.35 | <0.05 |
| Macrophages | - | - |  | 87.09±12.11 | 83.50±18.89 | ns |
| WBC (G/l) | 5.22±2.29 | 6.34±3.34 | ns |  |  |  |
| **Stable** | **Control**  **(n=10)** | **Alemtuzumab**  **(n=60)** | **p** | **Control**  **(n=10)** | **Alemtuzumab**  **(n=60)** | **p** |
| Lymphocytes | 1.28±0,57 | 0.569±0.406 | <0.001 | 6.32±12.07 | 5.44±9.67 | ns |
| Eosinophils | 0.061±0.038 | 0.081±0.111 | ns | 0.402±0.900 | 0.471±1.330 | ns |
| Neutrophils | 3.65±1.58 | 5.14±3.12 | ns | 2.90±5.62 | 5.96±9.41 | ns |
| Macrophages | - | - |  | 90.37±12.50 | 88.13±14.01 | ns |
| WBC | 5.50±1.37 | 6.24±3.25 | ns |  |  |  |
| **ACR** | **Control**  **(n=6)** | **Alemtuzumab**  **(n=12)** | **p** | **Control**  **(n=6)** | **Alemtuzumab**  **(n=12)** | **p** |
| Lymphocytes | 1.64±0.48 | 0.67 ±0.49 | <0.01 | 7.83±8.05 | 9.58±12.11 | ns |
| Eosinophils | 0.092±0.064 | 0.173±0.280 | ns | 1.82±3.34 | 0.92±1.92 | ns |
| Neutrophils | 3.75±2.82 | 4.57±2.67 | ns | 4.62±5.67 | 14.16±19.46 | ns |
| Macrophages | - | - |  | 85.74±11.24 | 75.34±25.04 | ns |
| WBC | 5.91±2.87 | 6.53±2.77 | ns |  |  | - |
| **LRTI** | **Control**  **(n=7)** | **Alemtuzumab**  **(n=50)** | **p** | **Control**  **(n=7)** | **Alemtuzumab**  **(n=50)** | p |
| Lymphocytes | 1.03±0.38 | 0.596±0.531 | <0.05 | 9.67±9.32 | 7.79±11.60 | ns |
| Eosinophils | 0.046±0.028 | 0.095±0.157 | ns | 0.161±0.219 | 0.719±2.450 | ns |
| Neutrophils | 2.56±2.30 | 5.69±3.45 | <0.01 | 5.98±12.91 | 13.78±17.90 | ns |
| Macrophages | - | - | - | 84.19±12.12 | 77.71±23.40 | ns |
| WBC | 3.95±2.62 | 6.81±3.74 | <0.05 |  |  |  |

**Table S1.** Comparison of peripheral blood mononuclear cell (PBMC) counts and bronchoalveolar lavage fluid immune cell (BALIC) percentages with or without alemtuzumab induction therapy in lung transplant recipients and in subgroups of patients exhibiting infection or rejection. WBC: white blood cell count.
