# Supplementary material for: Peripheral blood and bronchoalveolar leukocyte profile in lung transplant recipients and their changes according to immunosuppressive regimen: A single‐center experience
Source: Immun Inflamm Dis. 2022 Jul 12;10(8):e673. doi: 10.1002/iid3.673 (PMC9274796; doi:10.1002/iid3.673)
Supplement: Supplementary file 2 — Supporting Information. [file IID3-10-e673-s002.docx]

|  | **P_1-3_**  **n=26** | **P_4-6_**  **n=11** | **P_7-13_**  **n=23** |
| --- | --- | --- | --- |
| **PBMC**  WBC (G/L)  Neutrophils (G/L)  Lymphocytes (G/l)  Monocytes (G/l)  Eosinophils (G/l)  Basophils (G/L)  Neutrophils (%)  Lymphocytes (%)  Monocytes (%)  Eosinophils (%)  Basophils (%) | 5.90±4.62  5.31±4.44  0.216±0.109  0.312±0.204  0.051±0.055  0.009±0.027  87.76±6.61  4.71±3.13  6.20±3.59  1.18±1.22  0.152±0.227 | 5.56±2.39  4.46±1.96  0.494±0.242^*^  0.512±0.424  0.083±0.034  0.007±0.011  78.82±9.15^*^  9.97±4.47^**^  8.91±4.37  2.20±2.78  0.100±0.200 | 7.27±2.53  5.74±2.74  0.979±0.376^## ××^  0.459±0.204  0.086±0.105  0.005±0.005  76.23±11.41^##^  15.40±8.32^##^  6.85±3.04  1.45±1.87  0.069±0.085 |
| **BALIC**  BALMC (10^6^)  Macrophages (1/μl)  Neutrophils (1/μl)  Lymphocytes (1/μl)  Eosinophils (1/μl)  Macrophages (%)  Neutrophils (%)  Lymphocytes (%)  Eosinophils (%) | 12.85±7.04  11.15±6.54  0.768±0.890  0.222±0.314  0.036±0.073  83.33±18.05  7.48±11.51  1.68±1.44  0.238±0.584 | 14.58±5.71  11.41±6.79  0.563±0.630  0.442±0.306*  0.060±0.111  71.89±28.30  3.83±3.07  3.17±2.05  0.500±0.646 | 14.79±7.84  12.49±7.67  0.558±0.596  0.545±0.572  0.055±0.104  82.11±13.18  3.96±3.59  3.77±3.75  0.500±0.935 |

**Table S2.** Differences in PBMC and BALIC according to postoperative time periods in stable patients with alemtuzumab induction therapy. P_1-3_ lasted from the 14^th^ day until the end of the 3^rd^ month, P_4-6_ from the 4^th^ to the end of the 6^th^ month and P_7-13_ from the 7^th^ to the 13^th^. n: number of the patients, * p<0.05 and ** p<0.01 P_1-3_ vs P_4-6_, #p<0.05 and ## p<0.01 P_1-3_ vs P_7-13_, × p<0.05 and ×× p<0.01 P_4-6_ vs P_7-13_. BALMC: bronchoalveolar lavage fluid mononuclear cell count.
